# Comparisons of diazotrophic communities in native and agricultural desert ecosystems reveal plants as important drivers in diversity

**DOI:** 10.1093/femsec/fiv166

**Published:** 2015-12-24

**Authors:** Martina Köberl, Armin Erlacher, Elshahat M. Ramadan, Tarek F. El-Arabi, Henry Müller, Anastasia Bragina, Gabriele Berg

**Affiliations:** 1 Institute of Environmental Biotechnology, Graz University of Technology, 8010 Graz, Austria; 2 Faculty of Agriculture, Ain Shams University, 11566 Cairo, Egypt; 3 Biotechnology Laboratory, Heliopolis University, 11777 Cairo, Egypt

**Keywords:** desert farming, diazotrophs, medicinal plants, nitrogen-fixing communities, organic agriculture, *Rhizobiales*

## Abstract

Diazotrophs provide the only biological source of fixed atmospheric nitrogen in the biosphere. Although they are the key player for plant-available nitrogen, less is known about their diversity and potential importance in arid ecosystems. We investigated the nitrogenase gene diversity in native and agricultural desert soil as well as within root-associated microbiota of medicinal plants grown in Egypt through the combination of *nifH*-specific qPCR, fingerprints, amplicon pyrosequencing and fluorescence *in situ* hybridization–confocal laser scanning microscopy. Although the diazotrophic microbiota were characterized by generally high abundances and diversity, statistically significant differences were found between both soils, the different microhabitats, and between the investigated plants (*Matricaria chamomilla* L., *Calendula officinalis* L. and *Solanum distichum* Schumach. and Thonn.). We observed a considerable community shift from desert to agriculturally used soil that demonstrated a higher abundance and diversity in the agro-ecosystem. The endorhiza was characterized by lower abundances and only a subset of species when compared to the rhizosphere. While the microbiomes of the *Asteraceae* were similar and dominated by potential root-nodulating rhizobia acquired primarily from soil, the perennial *S. distichum* generally formed associations with free-living nitrogen fixers. These results underline the importance of diazotrophs in desert ecosystems and additionally identify plants as important drivers in functional gene pool diversity.

## Abstract (precis)

The diazotrophic microbiome of desert ecosystems is characterized by a high diversity and abundance and specific for each plant rhizosphere.

## INTRODUCTION

Nitrogen is one of the most yield-limiting factors in agricultural production systems throughout the world and an essential macronutrient for plants. Nitrogen-fixing microorganisms provide the only natural source of fixed atmospheric nitrogen in the biosphere (Gaby and Buckley 2012), and the capability for nitrogen fixation is widely dispersed among prokaryotic taxa including very divergent, distantly related bacteria and archaea (Zehr and Turner 2001; Zehr *et al.*^2003^). Biological nitrogen fixation by diazotrophic bacteria together with the input of recycled organic waste, such as manure or compost, is considered a sustainable alternative to chemical nitrogen fertilizers and a possibility to reduce rates of inorganic fertilizer application (Yang, Kloepper and Ryu 2009). The dispersal of inorganic nitrate into surface and groundwater often leads to eutrophication and severe environmental and health problems, yet can be largely avoided through biological inoculants (Orr *et al.*^2011^).

The *nifH* gene that encodes the nitrogenase reductase subunit is highly conserved over all nitrogenase types and has become the marker gene for studies of phylogeny, diversity and abundance of diazotrophic microorganisms (Zehr and Turner 2001). Phylogenetic analyses of *nifH* genes have revealed five primary clusters of homologous genes (Raymond *et al.*^2004^; Gaby and Buckley 2012). Although a wide range of environments has already been investigated for *nifH* gene diversity (López-Lozano *et al.*^2012^; Farnelid *et al.*^2013^; Yousuf *et al.*^2014^), the global census of diazotrophic diversity remains far from complete (Gaby and Buckley 2011, 2012). Next-generation sequencing techniques in combination with network analyses will allow a deeper insight into microbiome functioning involving nitrogen fixation.

Microbial viability and bioactivity play an important role in arid terrestrial ecosystems. Our previous research in desert ecosystems showed a higher indigenous antagonistic potential against soil-borne phytopathogens, a higher overall bacterial diversity, and better ecosystem function for plant health in soil used for desert agriculture in comparison to uncultivated native desert soil (Köberl *et al.*^2011^, ^2013^). Based on these results, we hypothesized (i) that the agricultural use of desert soil, especially crop rotation with leguminous cover crops, also enhances the diversity of the nitrogen-fixing underground communities. Plant specificity of associated bacteria as well as co-evolution of plant–microbe interactions are well-studied (Berg and Smalla 2009; Bulgarelli *et al.*^2012^; Oldroyd 2013), and root exudates are some of the most important drivers for this selecting effect from the soil microbiome (Bais *et al.*^2006^). Because we observed such a high specificity in the structural microbial diversity associated with medicinal plants (Köberl *et al.*^2013^), we hypothesized (ii) that this high specificity is visible in the functional *nifH* gene pool as well. All three selected plants for this study—German chamomile (*Matricaria chamomilla* L.), pot marigold (*Calendula officinalis* L.) and African nightshade (*Solanum distichum* Schumach. and Thonn.)—are well-known for their anti-microbial effects and bioactive ingredients (McKay and Blumberg 2006; Ukiya *et al.*^2006^; Bahgat *et al.*^2008^). Although the highly specific association between *Rhizobiaceae* and legumes is well investigated (Oldroyd 2013), less is known about the interactions of diazotrophic bacteria with non-legumes. In recent years, endophytic rhizobia were detected in some non-legumes as well, e.g. in rice, maize, barley, wheat, canola and lettuce, colonizing both the intercellular and intracellular spaces of epidermis, cortex and vascular system. They were associated with general plant growth promotion and improved grain yields in cereal crops (Yanni *et al.*^2001^; Chi *et al.*^2010^). Due to the extensive organic soil management, we hypothesized iii) that endophytic colonization with rhizobia occurs in non-leguminous plants cultivated on Sekem farms as well.

The objective of this research was to study the nitrogen-fixing communities in the endorhiza and rhizosphere of medicinal plants, and the bulk soil of long-term organically managed agricultural soil from the Sekem farms at Sharqia governorate in Egypt compared to unexploited native desert soil. We used a robust methodological approach combining *nifH*-specific qPCR, fingerprints and amplicon pyrosequencing. Complementary fluorescence *in situ* hybridization–confocal laser scanning microscopy (FISH–CLSM) analyses targeting the most dominant diazotrophic taxa were performed in order to reveal their habitat preferences and colonization type.

## MATERIALS AND METHODS

### Experimental design and sampling

Nitrogen-fixing communities were studied at the organically managed Sekem farm Adleya (www.sekem.com) located in the north-eastern desert region of Egypt near Bilbeis (30°13^′^44^″^N, 31°23^′^39^″^E) at Sharqia governorate. Physicochemical data of the soil is provided in Luske and van der Kamp (2009). The diazotrophic community of bulk agricultural soil was also investigated in comparison to the community of native desert soil from the Sinai Peninsula, Egypt (30°21^′^00^″^N, 32°15^′^18^″^E). At each site, four composite samples of soil in a horizon of 10–30 cm depth were collected. Profiles of the *nifH* gene in communities associated with the rhizosphere and endorhiza of three different species of medicinal plants (*M. chamomilla* L., *C. officinalis* L., and *S. distichum* Schumach. and Thonn.) cultivated on Adleya farm were studied and compared. From each plant species, four independent composite samples consisting of 5–10 plant roots with adhering soil were taken. The detailed sampling strategy is described by Köberl *et al.* (2011).

To isolate total community DNA from the soil and rhizosphere, 5 g of soil or roots with adhering soil were added to 45 mL of sterile 0.85% NaCl and vortexed. For isolation from the endorhiza, 5 g of roots were surface-sterilized with 4% NaOCl for 5 min. The roots were washed three times with sterile distilled water, then 10 mL sterile 0.85% NaCl were added and further homogenized using mortar and pestle. For isolation of total DNA from the rhizosphere, endorhiza and soil, 4 mL of the suspensions were centrifuged (16,000 × *g*, 4°C) for 20 min and the resulting microbial pellets were stored at –70°C. In the desert soil, a lower concentration of DNA was expected. Therefore, the pellets of 10 mL suspension were used for the isolation of total DNA. Total community DNA was extracted using the FastDNA SPIN Kit for Soil (MP Biomedicals, Solon, OH, USA).

### Quantification of microbial *nifH* genes by qPCR

To determine *nifH* gene abundances, quantitative PCRs were performed according to Hai *et al.* (2009) with following modifications. Reactions were conducted in a total volume of 10 μL containing 1× KAPA SYBR FAST qPCR MasterMix Universal (PEQLAB, Polling, Austria), 0.6 mg mL^−1^ BSA, 0.125 μM of primers nifH-F and nifH-R (Rösch, Mergel and Bothe 2002), and 0.8 μL template DNA dilutions with a concentration of ∼1 ng μL^−1^ (95°C, 10 min; 39 cycles of 95°C, 45 s; 55°C, 45 s; 72°C, 45 s; and melt from 72 to 95°C). Rotor-Gene 6000 real-time rotary analyzer (Corbett Research, Sydney, Australia) was used for fluorescence quantification. For absolute quantification, the PCR amplified *nifH* gene fragment from *Pectobacterium atrosepticum* SCRI1043 was ligated into the pGEM-T Easy Vector (Promega, Mannheim, Germany) and transformed into *Escherichia coli* DH5α. Serial dilutions of PCR fragments generated with the vector-specific primers USP and RSP (Köberl *et al.*^2011^) were used as standards for calculation of *nifH* gene copy numbers. Concentrations determined by absolute quantification were calculated to copy number per g soil or fresh weight (fw) of root. Each replicate sample was analyzed in duplicates in three independent runs. Statistical analysis was performed with PASW Statistics 18 (SPSS Inc., Chicago, IL, USA) using the independent samples *t*-test for differences between desert and agricultural soil, and the Games–Howell post hoc test for plant samples.

### Fingerprints from single-stranded conformational polymorphism analysis of the *nifH* gene

Fingerprinting of microbial communities using single-stranded conformational polymorphism was conducted as described by Schwieger and Tebbe (1998). Amplification of the *nifH* gene fragment was performed using a nested PCR approach with primer pairs nifH4/nifH3 (Zani *et al.*^2000^) and nifH11/nifH22^P^ (Yeager *et al.*^2004^). The obtained amplicons were separated and analyzed according to Bragina *et al.* (2012). Comparisons of SSCP generated *nifH* community profiles were performed using GelCompar II 5.1 (Applied Maths, Kortrijk, Belgium). The cluster analysis was performed with the following settings: dendrogram type: unweighted pair group method with arithmetic mean (UPGMA); similarity coefficient: curve based: Pearson correlation; position tolerances: optimization: 0%, position tolerance: 1%. Based on the Pearson similarity matrix, a multidimensional scaling (MDS) ordination plot was constructed. Pearson correlation matrices were additionally subjected to significance tests of pair-wise similarities by applying permutation analyses (*p* ≤ 0.05) using the permtest package of R statistics 2.13.1 (The R Foundation for Statistical Computing, Vienna, Austria) with 10^5^ random permutations of sample elements (Kropf *et al.*^2004^; R Development Core Team 2011).

### *nifH* gene profiling using 454 pyrosequencing

The nitrogenase gene *nifH* was amplified according to a nested PCR protocol with primers designed by Zani *et al.* (2000). The first PCR was performed with the primer pair nifH4/nifH3 as described above for SSCP analysis. Generated amplicons served as templates for the second PCR (30 μL) with the primer pair nifH1 and nifH2 designed by Zehr and McReynolds (1989) that contained the 454 pyrosequencing adaptors, linkers, and sample-specific tags (Table S1). Accordingly, 3 μL template dilutions were added to 1× Taq&Go (MP Biomedicals, Solon, OH, USA), 1.5 mM MgCl_2_ and 0.2 μM of each primer (95°C, 5 min; 30 cycles of 95°C, 1 min; 65.5°C, 1 min; 72°C, 30 s; and elongation at 72°C, 5 min). PCR products were purified by employing the Wizard SV Gel and PCR Clean-Up System (Promega, Madison, WI, USA). For rhizosphere samples, PCR products of two independent PCR reactions were pooled. For soil samples, respective PCR products from four independent replicate samples were pooled from each habitat in equal volumes. Pyrosequencing read libraries were sequenced by Eurofins Genomics (Ebersberg, Germany) using the Roche 454 GS-FLX+ Titanium sequencing platform. The nucleotide sequences are available in the European Nucleotide Archive (www.ebi.ac.uk/ena) under the BioProject accession number PRJEB10243.

Primer sequences were cropped, reads with low quality (minimum average base quality score 20) and a read length shorter than 200 nucleotides were removed, and remaining sequences were translated into their amino acid sequence using the tool FrameBot of RDP's FunGene pipeline (Fish *et al.*^2013^; Wang *et al.*^2013^). All subsequent analyses were carried out based on amino acid sequence datasets that were normalized to the same number of sequences within a habitat (5217 sequences per soil sample and 553 sequences per rhizosphere sample) using Subsetify 1.4 (Bragina *et al.*^2013^). Amino acid sequences were aligned and clipped at the same alignment reference position (∼108 amino acids) by using ClustalX 2.1 (Larkin *et al.*^2007^). OTUs were classified and rarefaction curves were constructed based on the distance matrices of amino acid sequences at 0%, 4% and 8% dissimilarity (Farnelid *et al.*^2011^) using mcClust and rarefaction of RDP's FunGene pipeline. Diversity indices were ascertained based on the clustering data (Shannon 1997; Chao and Bunge 2002). Significant differences in diversity indices were calculated with PASW Statistics 18 using Tukey and Games–Howell post hoc tests, depending on the homogeneity of variances. Representative sequences at 8% dissimilarity were selected for the following taxonomic and phylogenetic analysis (Farnelid *et al.*^2011^) where clusters with less than 1% of relative abundance were not designated. Nearest relatives were retrieved using the search tool tblastn of the NCBI database.

A neighbor-joining tree with 100 bootstrap replications was created with the tools seqboot, protdist, neighbor, consense and fitch of PHYLIP 3.69 (Felsenstein 1989). The phylogenetic tree was visualized and edited in MEGA4 (Tamura *et al.*^2007^). A heatmap showing the number of sequences for each OTU was added. As an outgroup root, a partial sequence of the light-independent photochlorophyllide reductase subunit L (BchL) from *Chlorobaculum tepidum* (accession number AAG12203) was selected. Chlorophyllide reductases share a small but significant degree of similarity with NifH (Zehr and Turner 2001).

A profile clustering network analysis was performed in order to highlight single OTUs (8% dissimilarity) with considerable differences between the rhizospheres of the medicinal plants. The network analysis was carried out with OTUs exhibiting a mean read change between plants of more than 1% of the normalized dataset. If the ratio of mean OTU read numbers exceeded two, the OTUs were regarded as altered and assigned to the respective profile. Visualization of the network was carried out using Cytoscape 2.8.2 (Smoot *et al.*^2011^). Significant differences between medicinal plants were calculated with Metastats (White *et al.*^2009^). P values were computed using a combination of the nonparametric *t*-test, exact Fisher's test and the false discovery rate with 10^3^ permutations.

### FISH and CLSM

In order to unravel habitat preferences and the colonization type of potential diazotrophs *in situ*, we carried out FISH–CLSM approaches using a robust set of specific fluorescent probes. The root system of *M. chamomilla* was used as representative subject. FISH was carried out as described by Cardinale *et al.* (2008). In brief, root samples were washed once with phosphate-buffered saline and then fixed in 4% paraformaldehyde. For the detection of *Alphaproteobacteria*, the Cy5-labeled ALF968 probe (Loy *et al.*^2007^) was used. *Betaproteobacteria* were detected with the Atto488-labeled BET42a probe applied together with an unlabeled competitor probe to avoid unspecific hybridizations (Manz *et al.*^1992^). An equimolar mixture of Cy3-labeled EUB338, EUB338II, and EUB338III probes (Amann *et al.*^1990^; Daims *et al.*^1999^) was used for the detection of all bacteria. In an additional ternary staining approach to visualize *Rhizobiales* with a high coverage, EUB338-MIX (Cy3) and ALF968 (Alexa488) were applied together with a combination of Cy5-labeled RHIZ1244 (Thayanukul *et al.*^2010^) and RHIZ3r (Erlacher *et al.*^2015^) probes. As negative controls, non-sense FISH probes labeled with all three fluorochromes (NONEUB) (Wallner, Amann and Beisker 1993) were applied. For better contrast, plant tissue sections were additionally stained by calcofluor-white staining (0.15% in H_2_O, 15 min incubation). Micrographs were acquired with a Leica TCS SPE confocal laser scanning microscope (Leica Microsystems GmbH, Mannheim, Germany) using the oil immersion objectives Leica ACS APO 40.0 × 1.15 (183.33 μm × 183.33 μm) and ACS APO 63 × 1.30 (116.40 μm × 116.40 μm). The Z-step was 0.7 μm. Solid state lasers were used with 405, 488, 532, 635 nm excitation. The colors red, green, and blue were assigned to the fluorochromes Cy3, Cy5 and Atto/Alexa488, respectively. Imaris 7.0 (Bitplane, Zurich, Switzerland) was used for micrograph post-processing and the assembly of iso-surface renderings.

## RESULTS

### Abundances of *nifH* genes in different underground communities

**Figure 1.**
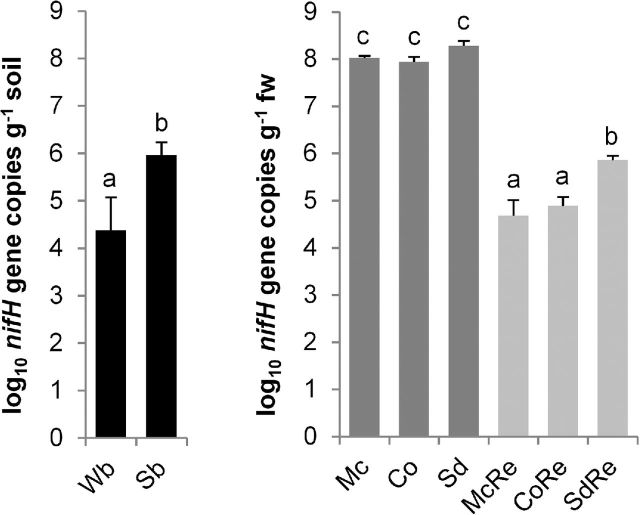
Abundances of *nifH* genes in bulk soils, rhizosphere, and endorhiza of medicinal plants detected by qPCR. Wb = desert soil, Sb = agricultural soil; Mc = *M. chamomilla*, Co = *C. officinalis*, Sd = *S. distichum*; Re = endorhiza, rhizosphere has no further designation. Averages of *nifH* gene copy numbers per gram soil or root fresh weight (fw) as log_10_ and confidences are shown. Significant differences between samples (*p* ≤ 0.05) are indicated by different letters.

Analysis of *nifH* gene copy numbers resulted in a statistically significant higher abundance in the agriculturally used soil (6.0 ± 0.3 log_10_ g^−1^) in comparison to the native desert soil (4.4 ± 0.7 log_10_ g^−1^; Fig. 1). Among the medicinal plant-associated microenvironments, rhizospheres showed significantly higher *nifH* gene copy numbers than endorhiza samples. Calculated abundances in the rhizospheres ranged from 7.9 ± 0.1 to 8.3 ± 0.1 log_10_ g^−1^ root fresh weight (fw) and were not significantly different between medicinal plants. Conversely, the endorhiza of the perennial *S. distichum* was more significantly colonized by nitrogen-fixing microorganisms (5.9 ± 0.1 log_10_ g^−1^ fw) than the endorhizas of the annual *Asteraceae M. chamomilla* (4.7 ± 0.3 log_10_ g^−1^ fw) and *C. officinalis* (4.9 ± 0.2 log_10_ g^−1^ fw; Fig. 1).

### Molecular fingerprinting of diazotrophic underground communities

SSCP fingerprints of *nifH* genes in microbial communities revealed the first insight into diversity (Fig. S1, Supporting Information). According to the statistical comparison analysis (Fig. S2, Supporting Information), the diazotrophic community composition of field soil differed significantly from the less diverse desert soil (*p* = 0.0286) by approximately 70%. Rhizospheres exhibited significantly different profiles from bulk agricultural soil (*p* = 0.0005) as well as from the inner tissue of the root (*p* < 0.0001). Also, statistically significant differences in nitrogen-fixing rhizosphere communities were detected between the three different medicinal plants (*p* values between 0.0285 and 0.0290). Approximately half of the *nifH* gene community was shared by all three plants; *M. chamomilla* and *C. officinalis* were more similar to each other, but still nearly 40% of their communities were determined by plant-specific nitrogen fixers (Fig. S2, Supporting Information). Dominant bands were identified as *Rhizobium*, *Bradyrhizobium* and *Burkholderia*. Furthermore, *Paenibacillus* spp. were assigned to dominant endorhiza bands. Throughout all plant-associated microenvironments, several cyanobacteria from the genera *Anabaena* and *Nostoc* were found. Additionally, the *nifH* gene sequence of the methanogenic archaeon *Methanocella* was identified (Fig. S1, Supporting Information).

### Pyrosequencing-based *nifH* profiling

A pyrosequencing-based analysis of the *nifH* gene was employed to gain a deeper insight into the diazotrophic community composition and diversity in soils and medicinal plant rhizospheres. Normalized NifH sequence datasets were rarefied at three cut-off levels (0%, 4% and 8% amino acid dissimilarity; Fig. S3, Supporting Information). Sequences from both soil types (10 434 reads) were classified into 361 OTUs with a dissimilarity cut-off of 8% (desert soil 118 OTUs; agricultural soil 290 OTUs), and rhizosphere sequences (6636 reads) were clustered into 400 OTUs (86–124 OTUs per sample). At a genetic dissimilarity level of 8%, the coverage of Chao1 estimated richness reached 61.1% and 56.9% for desert and agricultural soil, respectively (Table S2, Supporting Information). The calculated Shannon diversity index (H^′^) was much lower for the desert soil (H^′^ at a dissimilarity cut-off of 8% = 1.87) than for the agricultural soil (H^′^ = 3.92) indicating a higher diazotrophic diversity due to the agricultural use of the desert. The coverage of the rhizosphere samples was between 78.9 and 42.0%, and diversity indices showed no statistically significant differences (*p* ≤ 0.05) between plants; Shannon values ranged from 3.26 to 3.92 at 8% dissimilarity (Table S2, Supporting Information).

**Figure 2.**
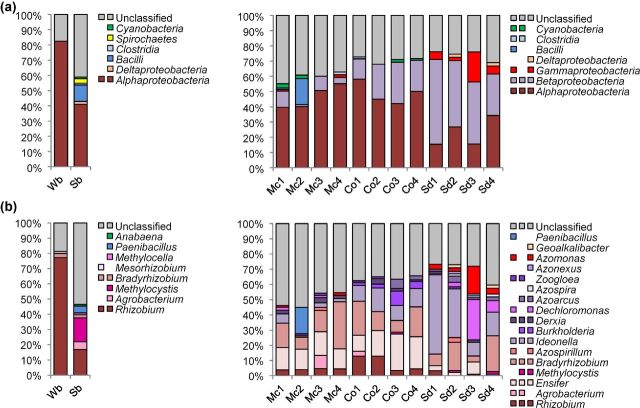
Taxonomic classification of NifH sequences in bulk soils and associated with the rhizosphere of medicinal plants obtained by 454 amplicon sequencing. Wb = desert soil, Sb = agricultural soil, Mc = *M. chamomilla*, Co = *C. officinalis*, Sd = *S. distichum*. NifH amino acid sequences were classified at class (**a**) and genus (**b**) level. From each medicinal plant, four independent replicate samples were investigated separately. PCR products of soil replicates were pooled to one composite sample per soil type. Clusters containing less than 1% of quality sequences were not designated. Multi-colored charts at the legend are shown for each soil or rhizosphere type correspondingly.

Among quality amino acid sequences, 70.6% of soil reads and 68.1% of rhizosphere reads could be taxonomically assigned to at least class level (Fig. 2). The desert soil in particular exhibited an overwhelming dominance of NifH sequences related to *Alphaproteobacteria* (82.4%). Field soil revealed a high proportion of unclassified sequences, yet still contained 41.0% *Alphaproteobacteria*. In regards to phyla with greater than 1% of quality reads: 1.9% of field soil sequences could be affiliated to *Deltaproteobacteria*, 10.8% to *Bacilli*, 1.2% to *Clostridia*, 2.8% to *Spirochaetes* and 1.1% to *Cyanobacteria*. Rhizosphere samples also revealed a high abundance of *Alphaproteobacteria* (58.0%–15.4%). Additionally, *Betaproteobacteria* were found in all samples (55.7%–1.3%). Higher proportions of *Alphaproteobacteria* were found in the rhizospheres of the *Asteraceae* (*M. chamomilla* 55.2%–39.8% and *C. officinalis* 58.0%–42.1%) in comparison to *Betaproteobacteria* which were more dominant in the rhizosphere of *S. distichum* (55.7%–27.1%). *Gammaproteobacteria* were additionally found in the rhizosphere of *S. distichum* (19.7%–2.4%) and *M. chamomilla* (≤1.8%), yet *Deltaproteobacteria* were only identified in the *S. distichum* rhizosphere (≤2.2%). Among *Firmicutes*, *Bacilli* were found associated with *M. chamomilla* (≤17.2%), and *Clostridia* with *M. chamomilla* (≤1.6%) and *C. officinalis* (≤1.3%). *Cyanobacteria* were found in the rhizospheres of *M. chamomilla* (≤2.7%) and *C. officinalis* (≤1.8%).

Representative sequences of the dominant alphaproteobacterial NifH clusters in native desert soil showed 99%–100% similarity to the NifH sequences of the genus *Rhizobium* (Table S3, Supporting Information). Further, *Bradyrhizobium* and *Mesorhizobium* were also found. The diversity within *Alphaproteobacteria* was higher in the agriculturally used soil with 40.9% identified as *Rhizobium*, 12.9% as *Agrobacterium*, 38.2% as *Methylocystis*, 5.1% as *Bradyrhizobium* and 2.9% as *Methylocella*. Classifiable *Bacilli* in unplanted field soil were identified as *Paenibacillus*, and all cyanobacterial reads revealed the genus *Anabaena* as the closest hit. *Deltaproteobacteria*, *Clostridia* and *Spirochaetes* could not be identified at the genus level (<95% of sequence similarity). In all three investigated medicinal plant rhizospheres, alphaproteobacterial sequences of the genera *Rhizobium*, *Ensifer* and *Bradyrhizobium* were found, and *Agrobacterium* was identified in the rhizospheres of the *Asteraceae*. *Methylocystis* was found associated with roots of *C. officinalis* and *S. distichum*, and *Azospirillum* with *M. chamomilla* and *S. distichum*. Clusters of *Betaproteobacteria* found in all rhizospheres were identified as *Ideonella* and *Derxia*, and *Zoogloea* was found associated with both *Asteraceae*. In the rhizospheres of *C. officinalis* and *S. distichum*, nitrogen-fixing *Burkholderia* and *Azoarcus* were additionally identified. *Dechloromonas* was found in rhizospheres of *M. chamomilla* and *S. distichum*, and *Azospira* and *Azonexus* were only associated with the *S. distichum* root. Classifiable *Gammaproteobacteria* in the rhizospheres of *M. chamomilla* and *S. distichum* were affiliated with the genus *Azomonas*. All deltaproteobacterial reads found associated with *S. distichum* were classified in the genus *Geoalkalibacter*, and all classifiable *Bacilli* reads in the rhizosphere of *M. chamomilla* were identified as *Paenibacillus*.

**Figure 3.**
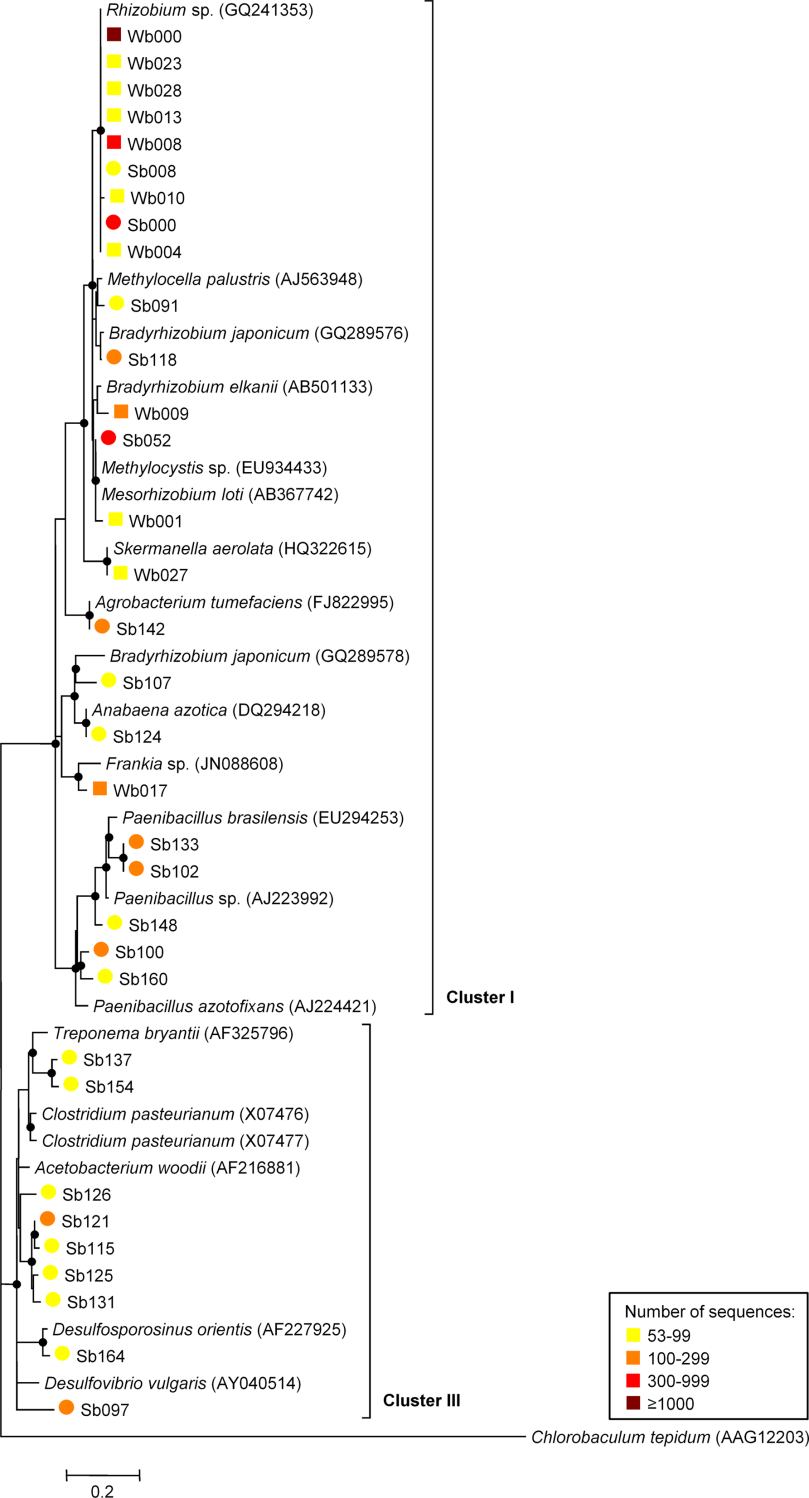
Phylogenetic composition of diazotrophic communities in desert (Wb, squares) and agricultural soil (Sb, circles). Each dataset comprised 5217 high-quality NifH sequences clustered at 8% dissimilarity. The neighbor-joining phylogenetic tree was constructed with both one representative sequence per each OTU and the closest database match (accession numbers in brackets). A partial sequence of the light-independent photochlorophyllide reductase subunit L (BchL) from *C. tepidum* (AAG12203) was used as an outgroup. Reliability of the tree topology was evaluated using bootstrap analysis with 100 re-samplings (bootstrap values > 50% are indicated as black circles). Sequences were affiliated to the canonical *nifH* clusters I and III. Numbers of sequences in each cluster are indicated in a heatmap. OTUs containing less than 1% of the normalized dataset were not phylogenetically designated. The scale bar indicates 0.2 amino acid substitutions per site.

In a phylogenetic tree distinguishing between the major *nifH* gene types, all NifH sequences from the soil sample libraries were affiliated with the canonical *nifH* clusters I and III (Fig. 3). Conventional molybdenum nitrogenases (cluster I) were dominated by *Alphaproteobacteria* (closest related to *Rhizobium*, *Methylocella*, *Bradyrhizobium*, *Methylocystis*, *Mesorhizobium*, *Skermanella* and *Agrobacterium*), but also contained sequences from *Cyanobacteria* (*Anabaena*), *Actinobacteridae* (*Frankia*) and *Bacilli* (*Paenibacillus*). Reads affiliated with the conventional molybdenum nitrogenases from anaerobes (cluster III) were most closely related to *Spirochaetes* (*Treponema*), *Clostridia* (*Clostridium*, *Acetobacterium* and *Desulfosporosinus*) and *Deltaproteobacteria* (*Desulfovibrio*). No sequences of alternative nitrogenases (cluster II) and *nifH* paralogs (clusters IV and V) were found in the soil libraries.

**Figure 4.**
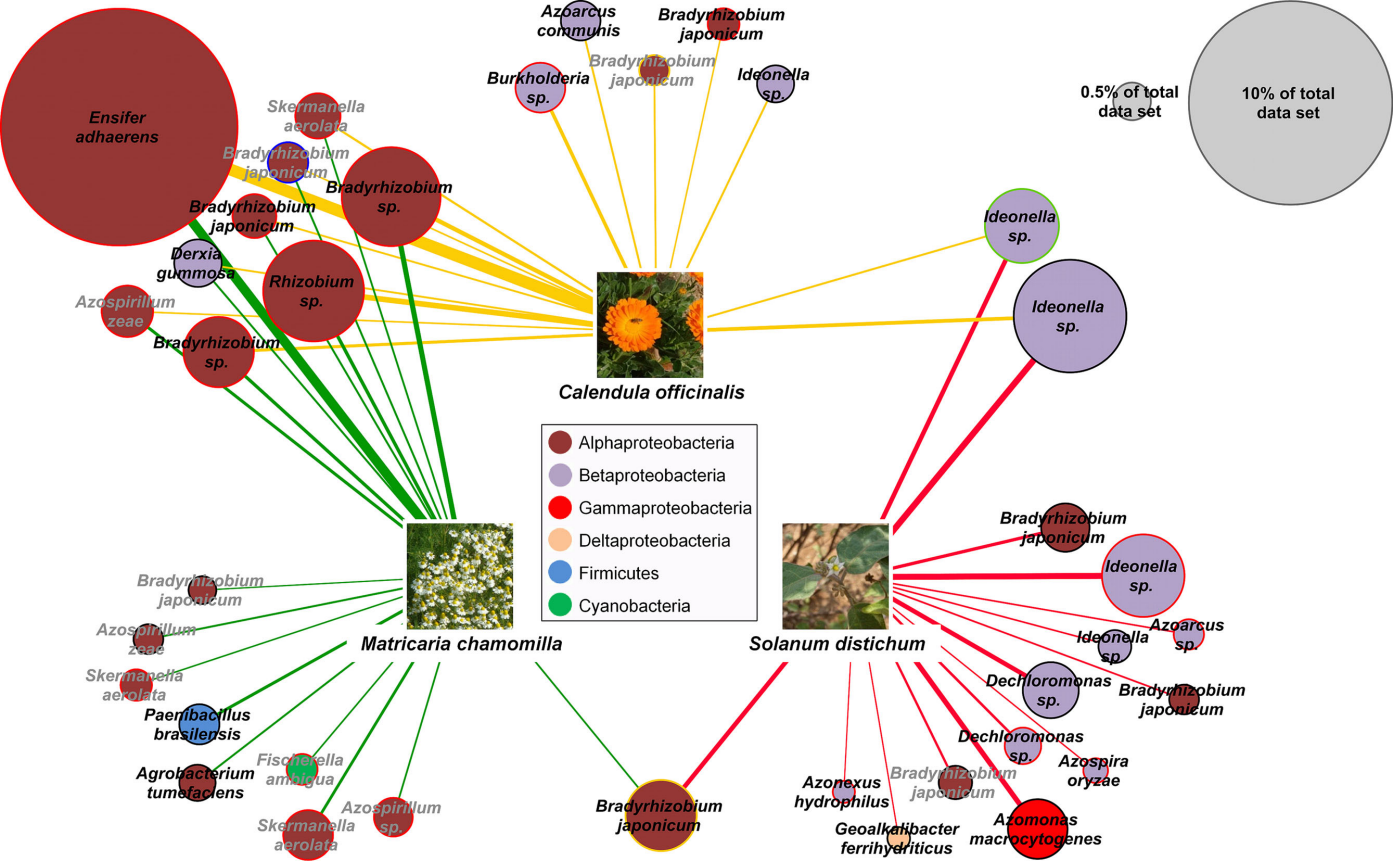
Profile clustering network analysis of NifH sequence libraries of rhizosphere samples from *M. chamomilla*, *C. officinalis* and *S. distichum* at a dissimilarity level of 8%. The abundance values for OTUs with a mean read change between plants of more than 1% of the normalized dataset were used. If the ratio of mean OTU read numbers exceeded 2, the OTUs were regarded as altered and assigned to the respective profile. Node sizes of OTUs correspond to the relative abundance of the total dataset; nodes matching abundances of 0.5% and 10% were added as reference points. Distributions between plants are displayed by widths of connection lines. Significances (*p* ≤ 0.05) are indicated by colored node borders: red node borders indicate significances between connected and all not linked profiles, green is used for significances between *Matricaria* and *Calendula*, orange for significances between *Calendula* and *Solanum*, blue for significances between *Matricaria* and *Solanum*, and nodes with black borders for no significant differences. Black node labels indicate a similarity to the taxonomic node label (closest database match) of ≥ 95%, whereas gray node labels have a similarity < 95%.

A profile clustering network analysis was applied to achieve better insight into the differences between the diazotrophic communities of the three medicinal plant rhizospheres (Fig. 4). OTUs equally distributed among all three rhizospheres were neglected in this network. The rhizosphere profiles revealed more similar diazotrophic communities between *M. chamomilla* and *C. officinalis* which were dominated by potential root nodule bacteria. NifH sequences of OTUs identified as genera *Ensifer*, *Rhizobium* and *Bradyrhizobium* were found in significantly higher abundances in the rhizospheres of *Asteraceae*. Conversely, the rhizosphere of *S. distichum* was colonized in greater numbers by nitrogen-fixing *Beta-*, *Gamma-* and *Deltaproteobacteria*. Significantly higher read counts were obtained for OTUs identified as *Ideonella*, *Dechloromonas*, *Azoarcus*, *Azospira* and *Azonexus*. For each medicinal plant, several specific OTUs were found within the nitrogen-fixing community.

### *In situ* visualization of bacterial rhizosphere colonization

**Figure 5.**
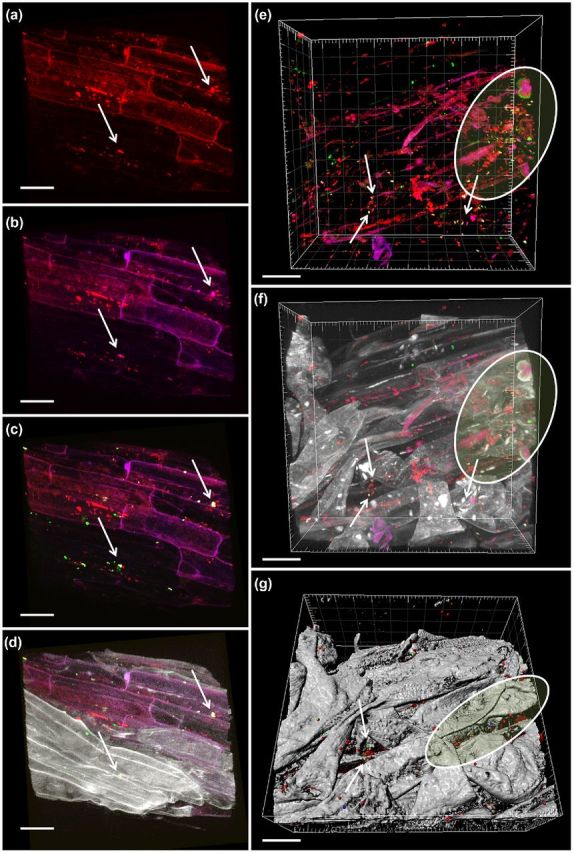
*In situ* visualization of bacterial colonization in the root system of *M. chamomilla*. Volume renderings (**a**–**f**) and three-dimensional reconstruction model made by Imaris (**g**) of confocal laser scanning microscopy stacks. Micrographs a–d show the efficacy of the FISH-probes and potential endo- and ectophytic root colonization patterns of *Rhizobiales* (white arrows). Panels e–g show mixed bacterial taxa including *Rhizobiales* and other *Alphaproteobacteria* colonizing niches (white marks) and the inner compartments of the root. white/green (g) = *Rhizobiales*; pink/blue (g) = *Alphaproteobacteria*; red = all bacteria; gray = root tissue. Scale bars = 20 μm. Probe set: RHIZ1244 (Cy5), RHIZ3r (Cy5), ALF968 (Alexa488), EUB338-MIX (Cy3). The colors red, green, and blue were assigned to the fluorochromes Cy3, Cy5 and Alexa488, respectively.

Complementary FISH–CLSM analyses revealed generally strong rhizoplanic colonization. High abundances of *Alphaproteobacteria* and in particular the order *Rhizobiales* were observed in the root systems of medicinal plants grown under arid desert farming conditions (Fig. 5; S4, S5, Supporting Information). *Alphaproteobacteria* were, among the root-associated bacteria, clearly dominant over *Betaproteobacteria* and were found as both single cells and in large colonies in close interaction with other undefined bacteria (Fig. S4, Supporting Information). A high proportion of *Alphaproteobacteria* were subsequently identified as *Rhizobiales* (Fig. 5). As suggested in Erlacher *et al.* (2015), the combination of the FISH probes RHIZ1244 and RHIZ3r yielded into a robust coverage of most *Rhizobiales* including the predominant taxa *Rhizobiaceae* and *Bradyrhizobiaceae*. The tendency of *Rhizobiales* to preferentially form small sub-clusters consisting of three to six cells prevailed over the participation in bacterial plaques or biofilm like structures (Fig. 5). We observed both, packed and dense colonization on the rhizoplane and bacteria in the endorhiza. Further, *Rhizobiales* were found predominantly in niches of the rhizoplane and in compartments of the endorhiza.

## DISCUSSION

We observed a considerable shift in the diazotrophic soil community towards both higher abundance and diversity after long term organic desert agriculture, thus supporting our first hypothesis. The agricultural use of desert soil, especially through crop rotation with leguminous cover crops such as alfalfa or clover, enhances the presence and diversity of the nitrogen-fixing underground community. Nitrogen-fixing microorganisms in deserts play an indispensable role for both plant development and growth, yet desert soils are characterized by harsh environmental conditions, e.g. extreme temperatures, desiccation, high soil salinity, low nutrient levels, high UV radiation levels and physical instability caused by strong winds (Cary *et al.*^2010^) which can all be transformed into a more manageable environment by watering and cultivating plants. Native desert soil, however, still showed an impressive diversity and relatively high abundance in comparison to other ecosystems. Contrary to previous studies that describe desert microbial communities as solely structured through abiotic processes (Cary *et al.*^2010^), we additionally identified plants as important drivers for the functional gene pool diversity in arid soil ecosystems.

As stated in our second hypothesis, the high specificity for each plant species is in a functional gene. While specific structures of microbial communities were previously described (Berg and Smalla 2009), this specificity was confirmed for the structure of *Arabidopsis* cultivars using amplicon libraries (Bulgarelli *et al.*^2012^). In this study, we found evidence of the specificity of the diazotrophic communities associated with the three plant species. In general, diazotrophic communities in the medicinal plant-associated microenvironments (rhizosphere and endorhiza) were more similar between the two *Asteraceae* in comparison to the *Solanum*, and the *nifH* gene profiles revealed a higher abundance of *Alphaproteobacteria* associated with the roots of the *Asteraceae*. Conversely, the nightshade showed a higher proportion of *Betaproteobacteria*. Similarly, previous investigations of the total bacterial and fungal communities already revealed comparable colonization patterns between *M. chamomilla* and *C. officinalis* in comparison to *S. distichum* (Köberl *et al.*^2013^). Both *Asteraceae* are well-known medicinal plants that produce more similar bioactive metabolites, including several flavonoids, which could explain their similar microbiomes. The bioactive ingredients of the African *S. distichum* were all not yet identified (Bahgat *et al.*^2008^). Furthermore, *M. chamomilla* and *C. officinalis* are annual herbal medicinal plants, while *Solanum distichum* is a perennial plant, thus providing a longer timeframe to specifically select a stable associated microbiome.

Nitrogen-fixing alphaproteobacterial genera were also largely represented in both soils where plants primarily acquire their associated microbial community (Berg and Smalla 2009). The question remains, however, as to where the *Beta-* and *Gammaproteobacteria* originated as they were identified neither in the desert nor in agriculturally conditioned bulk soil. Transmission through seeds or pollen would be possible, as recent studies on pumpkin flowers showed that the pollen grain surfaces are densely colonized by *Beta-* and *Gammaproteobacteria* (Fürnkranz *et al.*^2012^). Even though plant seeds are well-known carriers of seed-borne pathogens, they can also transmit beneficial microorganisms (Hardoim *et al.*^2012^). A very rare abundance of *Beta-* and *Gammaproteobacteria* within diazotrophic communities of bulk arid soils combined with a recruitment by root exudates and the favorable conditions of plant rhizospheres could explain this phenomenon as well.

In general, all microenvironments were primarily inhabited by proteobacterial nitrogen fixers, and the desert soil was noticeably dominated by *Rhizobiales*. Similar results were reported by López-Lozano *et al.* (2012) for soil from the Chihuahuan Desert in Mexico investigated using *nifH* gene clone libraries. However through the use of deep sequencing, we detected a much higher diversity: NifH sequences from soils and rhizosphere reads could be classified at a genetic dissimilarity level of 8% into 361 OTUs and 400 OTUs, respectively. Moreover, pyrosequencing techniques for *nifH* gene sequencing allow for the analysis of more sequences than those analyzed for the global study published a few years before (Gaby and Buckley 2011). The high NifH diversity primarily encompasses bacteria, as the only suggestion for archaeal nitrogen fixation in these unique desert habitats was found with *Methanocella* in the endorhiza, especially from *C. officinalis*. The *nifH* genes are commonly found in many methanogens, but not all methanogens are capable of nitrogen fixation. A complete *nif* operon that enables the organism to undergo nitrogen fixation was recently detected in the genome of *Methanocella conradii* HZ254 (found as closest database match in this study) (Lü and Lu 2012), however the genome of the closely related species *M. paludicola* SANAE only contains genes similar to *nifH* that are neither part of an operon with other *nif* genes nor associated with the nitrogen fixation function (Sakai *et al.*^2011^). Through comparisons with the metagenomically reconstructed genome sequence of the Rice Cluster I archaeon RC-I_MRE50_ for which a full component of the genes for nitrogenase was found (Erkel *et al.*^2006^), Sakai *et al.* (2011) have already discussed an inter-species physiological difference between the nitrogen fixation capabilities among the members of the order *Methanocellales*.

With the same primer pair, we calculated much higher abundances of *nifH* genes in the rhizospheres of the investigated medicinal plants in Egypt than Hai *et al.* (2009) in the rhizosphere of *Sorghum bicolor* grown in Burkina Faso. Only a fraction of the medicinal plant-associated nitrogen fixers were able to invade the root, compete with other well-adapted root endophytes, and successfully colonize the inner tissue. Abundances calculated for endorhizas totaled 58%–71% of the appropriate rhizosphere, and the diversity of nitrogen-fixing microorganisms in the inner part of the root was noticeably lower. All habitats influenced by desert agriculture contained a higher amount of potential nitrogen fixers, whereby in particular the rotation with legume plants is a considerable source of rhizobia. The rhizobial life-cycle typically includes a nitrogen-fixing endosymbiosis within legume root-nodules and a free-living saprophytic persistence in soil; however, the detection of endosymbiotic life of rhizobia in non-legumes (Yanni *et al.*^2001^; Chi *et al.*^2010^) demonstrated that some rhizobia have evolved a three-component life-cycle including a beneficial plant growth-promoting endocolonization within non-legume roots when grown in rotation with leguminous hosts. As hypothesized, endophytic colonization by different rhizobial strains could also be shown for the investigated non-leguminous medicinal plant roots through *nifH*-specific fingerprints and *Rhizobiales*-specific FISH microscopy.

An immense diversity and high abundance of diazotrophic communities were detected in all investigated arid habitats, thus strongly supporting their important role in native and agricultural desert ecosystems. The uniform distribution of dominant *Rhizobiales* across plant roots and the frequent occurrence in the endorhiza also contributes to their important role as symbiotic partners for their plant hosts.

## Supplementary Material

Supplementary Data
