# Supplementary material for: Comparisons of diazotrophic communities in native and agricultural desert ecosystems reveal plants as important drivers in diversity
Source: FEMS Microbiol Ecol. 2015 Dec 24;92(2):fiv166. doi: 10.1093/femsec/fiv166 (PMC4730177; doi:10.1093/femsec/fiv166)
Supplement: Supplementary Data [file fiv166_supplementary_data.zip › Supporting_Information.pdf]

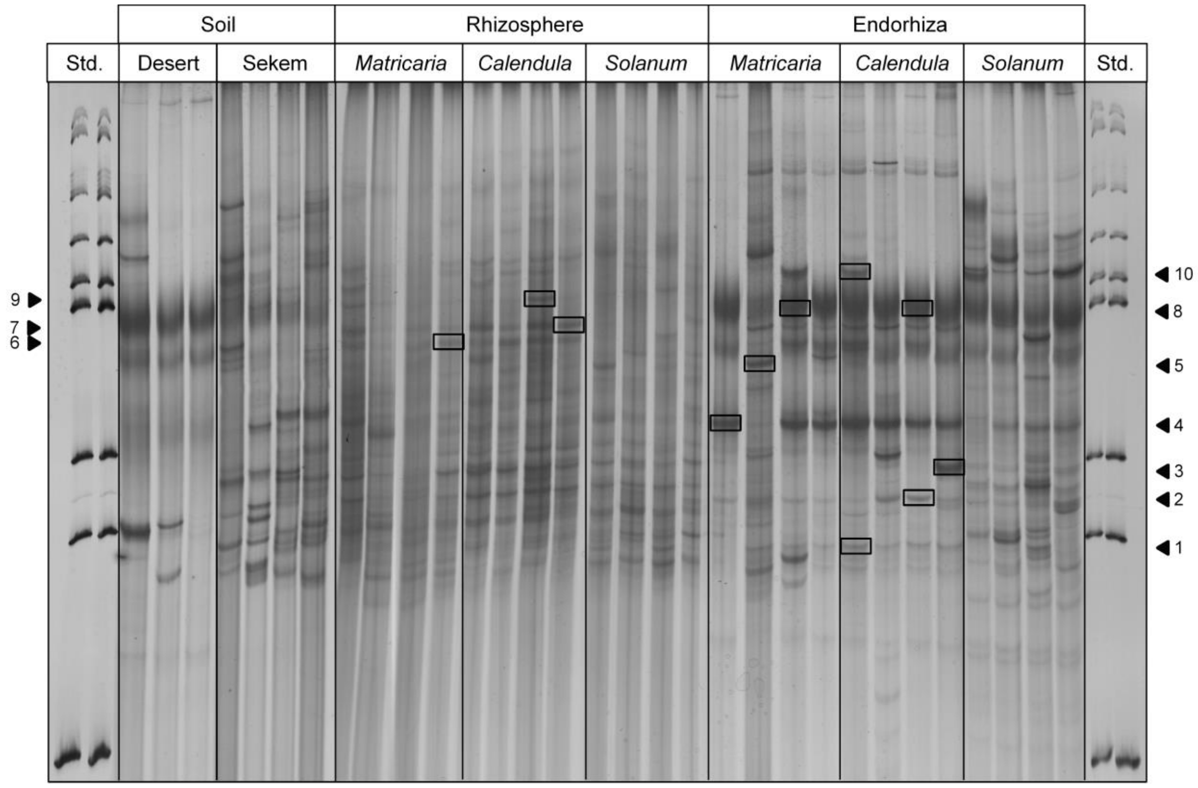

Fig. S1. *nifH* PCR-SSCP profiles from desert and agricultural soil as well as the rhizosphere and endorhiza of the medicinal plants *M. chamomilla*, *C. officinalis*, and *S. distichum*. Std.: 1 kb DNA ladder. Closest database matches of the following bands were identified: 1. *Bradyrhizobium japonicum*, 90% similarity to GenBank accession number GQ289576; 2. *Paenibacillus terrae*, 95% similarity to CP003107; 3. *Methanocella conradii*, 96% similarity to CP003243; 4. *Paenibacillus polymyxa*, 96% similarity to HM146187; 5. *Nostoc* sp., 93% similarity to BA000019; 6. *Nostoc punctiforme*, 90% similarity to CP001037; 7. *Anabaena* sp., 92% similarity to HQ836215; 8. *Rhizobium* sp., 95% similarity to FN666268; 9. *Anabaena* sp., 95% similarity to L04499; 10. *Burkholderia xenovorans*, 89% similarity to EF158805.

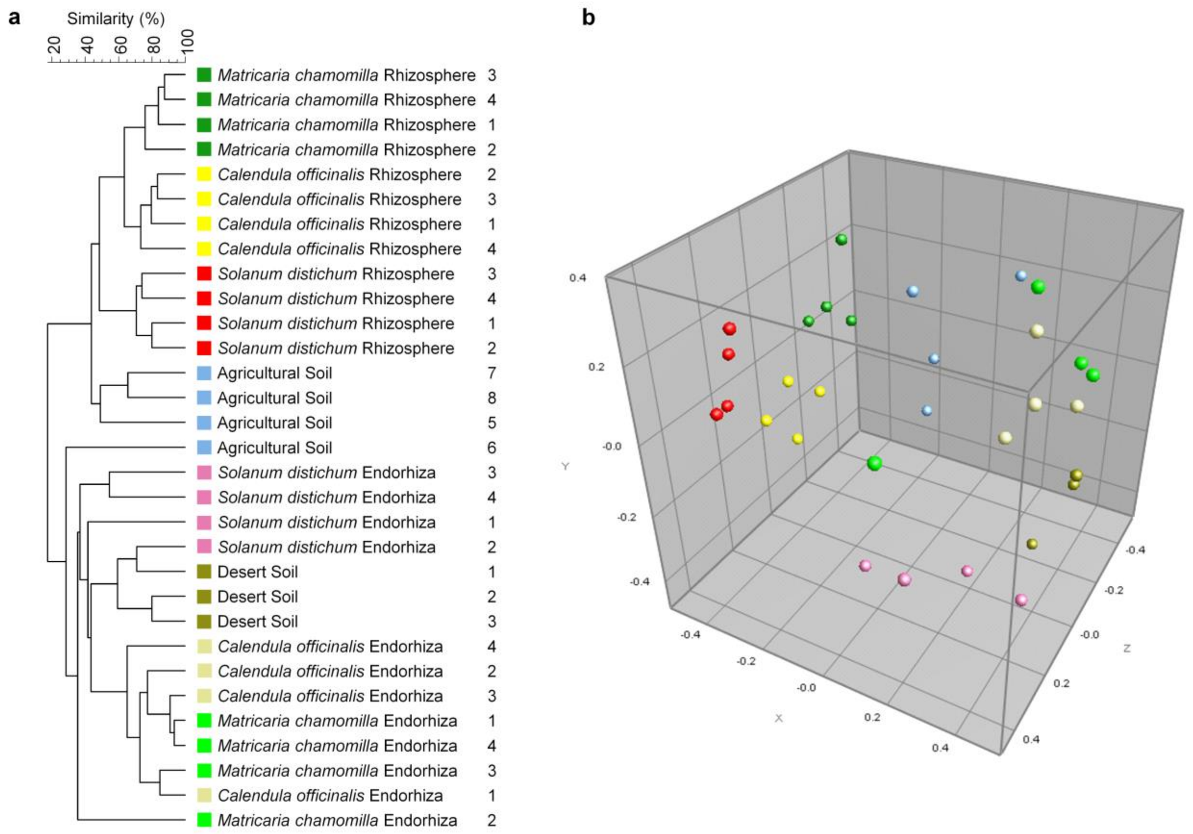

Fig. S2. Comparison analysis of PCR-SSCP profiles of the *nifH* gene patterns in bulk soil, rhizosphere, and endorhiza of the medicinal plants. (a) Unweighted pair group method with arithmetic mean (UPGMA) tree. Numbers indicate independent replicate samples. The dendrogram was generated with GelCompar II using Pearson correlation and an optimization of 0%. (b) Multidimensional scaling (MDS) ordination plot based on Pearson similarity matrix. Colors correspond to squares in the tree.

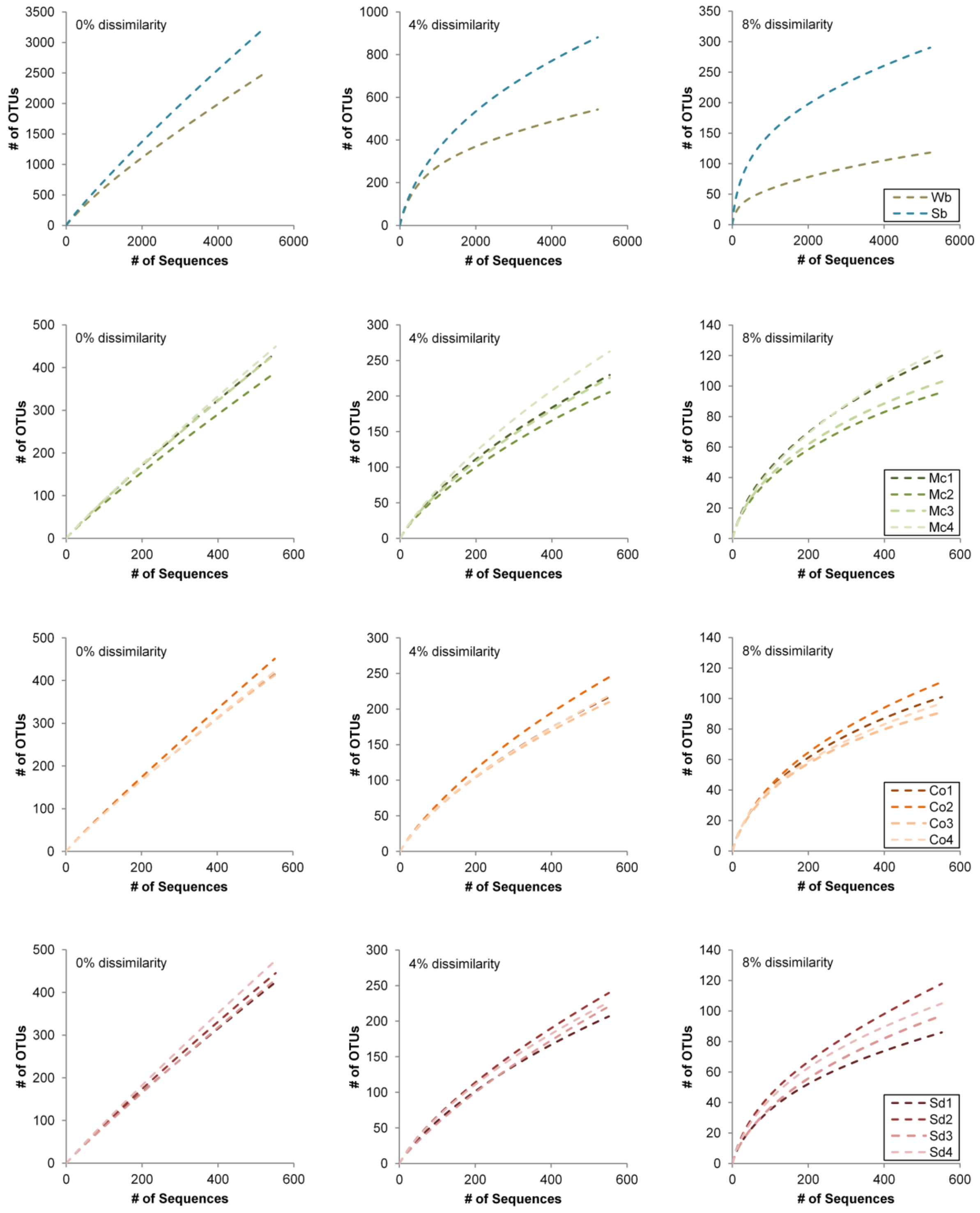

Fig. S3. Rarefaction analyses for NifH sequence datasets of bulk and rhizosphere soil samples. Wb = desert soil, Sb = agricultural soil, Mc = *M. chamomilla*, Co = *C. officinalis*, Sd = *S. distichum*. For the medicinal plants, curves are presented from four independent replicate samples per species. OTUs are shown at amino acid distance levels of 0%, 4%, and 8%.

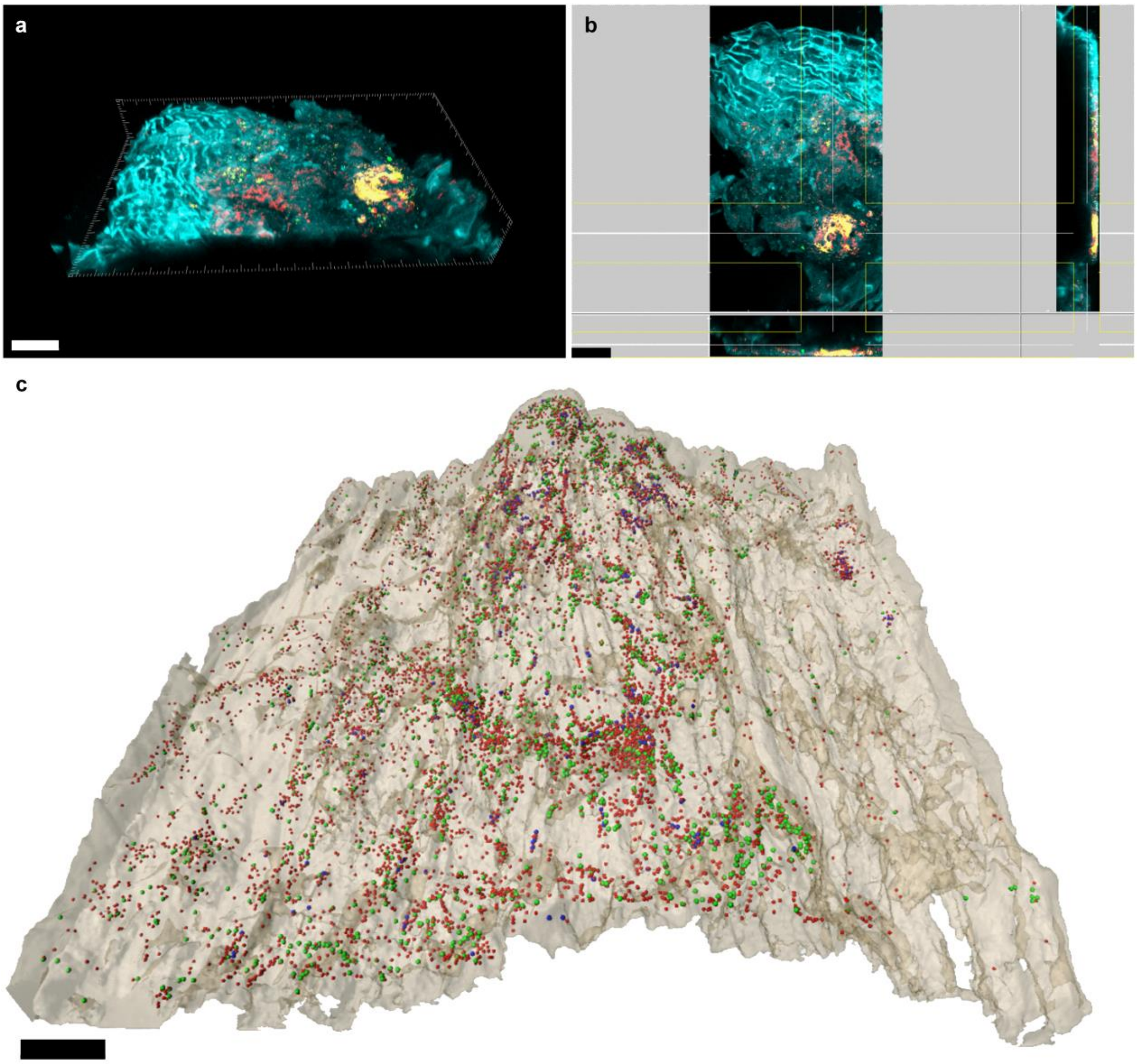

Fig. S4. *In situ* visualization of strong bacterial colonization in the root system of *M. chamomilla*. Volume rendering (a) and corresponding XY, XZ, YZ projections (b) of a biofilm like structure consisting mainly of *Alphaproteobacteria* on a tumor like root deformation. Three-dimensional reconstruction model made by Imaris (c) of confocal laser scanning microscopy stacks from the rhizoplane heavily colonized by bacteria. (a-b) yellow = *Alphaproteobacteria*, pink = *Betaproteobacteria*, red = other bacteria, cyan = root tissue (c) green = *Alphaproteobacteria*, blue = *Betaproteobacteria*, red = other bacteria, beige = root tissue. Scale bars = 30 µm. Probe set: ALF968 (Cy5), BET42a (Atto488), BET42a-competitor (unlabeled), EUB338-MIX (Cy3). The colors red, green, and blue were assigned to the fluorochromes Cy3, Cy5, and Atto488, respectively.

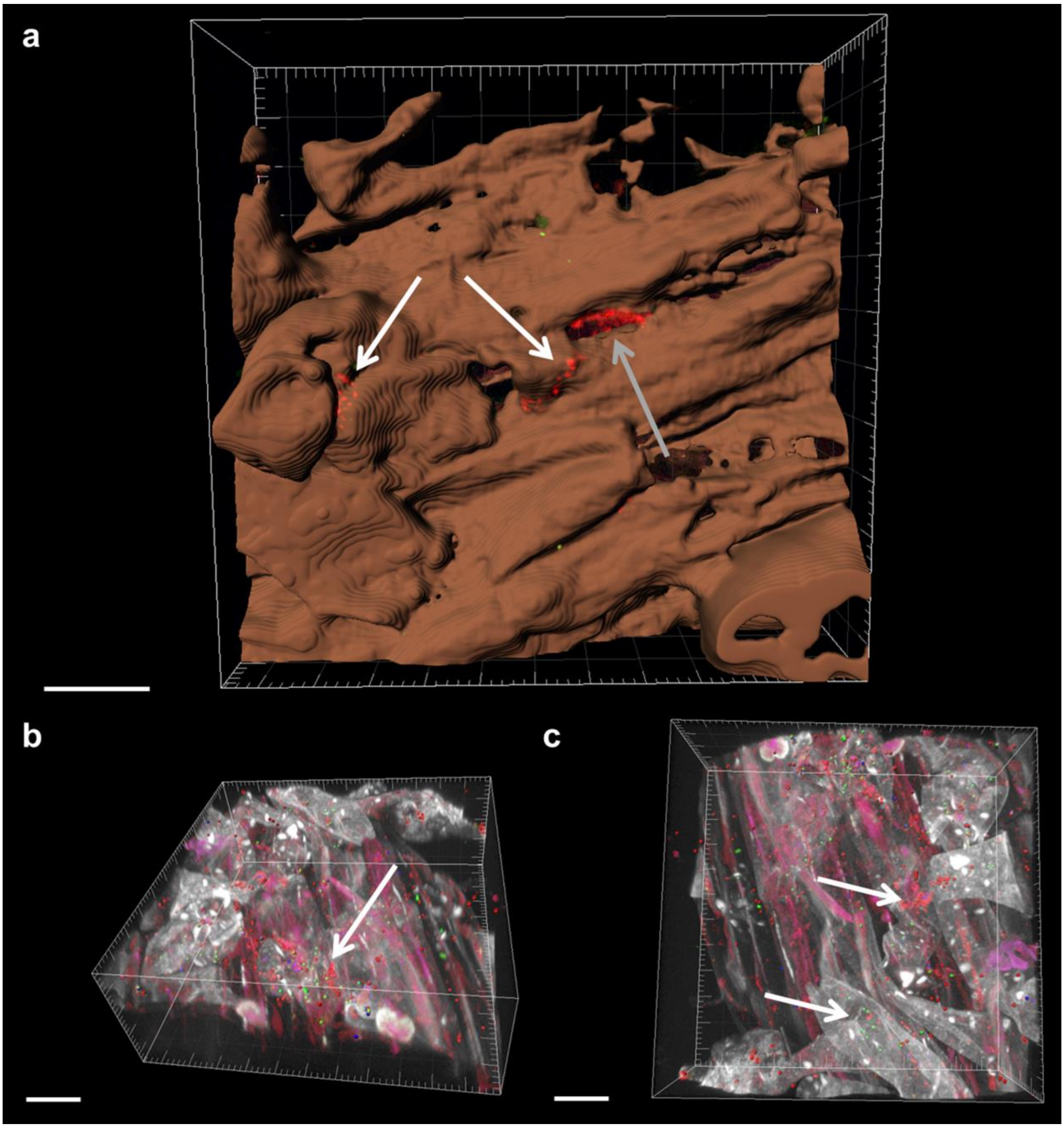

Fig. S5. *In situ* visualization of bacterial colonization in the root system of *M. chamomilla*. Three-dimensional reconstruction model made by Imaris (a) and volume renderings (b-c) of confocal laser scanning microscopy stacks show bacteria attached to a niche on the outer rhizoplane (grey arrow) and putative colonization of the endorhizal compartments of the root (white arrows). (a-c) green = *Rhizobiales*; blue = *Alphaproteobacteria*; red = other bacteria; brown (a), grey (b-c) = root tissue. Scale bars = 20 µm. Probe set: RHIZ1244 (Cy5), RHIZ3r (Cy5), ALF968 (Alexa488), EUB338-MIX (Cy3). The colors red, green, and blue were assigned to the fluorochromes Cy3, Cy5, and Alexa488, respectively.

Table S1. Custom primers including 454 pyrosequencing adaptors (bold), linkers (italic) and sample specific tags (underlined).

Name	Primer sequence
nifH1_MID21	**CGTATCGCCTCCCTCGCGCCA***TCAG*CGTAGACTAGTGYGAYCCNAARGCNGA
nifH1_MID22	**CGTATCGCCTCCCTCGCGCCA***TCAG*TACGAGTATGTGYGAYCCNAARGCNGA
nifH1_MID23	**CGTATCGCCTCCCTCGCGCCA***TCAG*TACTCTCGTGTGYGAYCCNAARGCNGA
nifH1_MID24	**CGTATCGCCTCCCTCGCGCCA***TCAG*TAGAGACGAGTGYGAYCCNAARGCNGA
nifH1_MID25	**CGTATCGCCTCCCTCGCGCCA***TCAG*TCGTCGCTCGTGYGAYCCNAARGCNGA
nifH1_MID26	**CGTATCGCCTCCCTCGCGCCA***TCAG*ACATACGCGTTGYGAYCCNAARGCNGA
nifH1_MID27	**CGTATCGCCTCCCTCGCGCCA***TCAG*ACGCGAGTATTGYGAYCCNAARGCNGA
nifH1_MID28	**CGTATCGCCTCCCTCGCGCCA***TCAG*ACTACTATGTTGYGAYCCNAARGCNGA
nifH1_MID29	**CGTATCGCCTCCCTCGCGCCA***TCAG*ACTGTACAGTTGYGAYCCNAARGCNGA
nifH1_MID30	**CGTATCGCCTCCCTCGCGCCA***TCAG*AGACTATACTTGYGAYCCNAARGCNGA
nifH1_MID31	**CGTATCGCCTCCCTCGCGCCA***TCAG*AGCGTCGTCTTGYGAYCCNAARGCNGA
nifH1_MID32	**CGTATCGCCTCCCTCGCGCCA***TCAG*AGTACGCTATTGYGAYCCNAARGCNGA
nifH2_454	**CTATGCGCCTTGCCAGCCCGC***TCAG*ADNGCCATCATYTCNCC

Table S2. Richness estimates and diversity indices obtained at 0%, 4%, and 8% amino acid dissimilarity for NifH sequence libraries of bulk soil and rhizosphere samples.

Sample[a]	Quality reads[b]	Clusters[c] (OTUs)			Chao1 (OTUs)			Coverage (%)			Shannon (H')		
		0%	4%	8%	0%	4%	8%	0%	4%	8%	0%	4%	8%
Wb	5,217	2,487	543	118	18,564	1,050	193	13.4	51.7	61.1	6.20	4.77	1.87
Sb	5,217	3,228	881	290	22,195	1,680	509	14.5	52.4	56.9	7.17	5.41	3.92
Mc1	553	435	230	120	3,990	613	186	10.9	37.5	64.5	5.84	4.77	3.92
Mc2	553	390	206	96	3,479	410	137	11.2	50.2	70.1	5.51	4.48	3.58
Mc3	553	434	226	103	3,834	558	155	11.3	40.5	66.6	5.82	4.68	3.70
Mc4	553	450	263	124	5,292	677	210	8.5	38.8	59.1	5.87	4.92	3.83
Co1	553	415	217	101	2,642	495	146	15.7	43.8	69.2	5.73	4.61	3.67
Co2	553	452	245	111	6,057	633	202	7.5	38.7	55.1	5.88	4.78	3.69
Co3	553	414	210	91	2,209	448	115	18.7	46.9	78.9	5.71	4.49	3.45
Co4	553	422	219	97	3,942	831	166	10.7	26.3	58.4	5.72	4.60	3.62
Sd1	553	425	207	86	4,417	421	168	9.6	49.2	51.2	5.73	4.44	3.26
Sd2	553	446	240	118	3,506	806	281	12.7	29.8	42.0	5.88	4.80	3.89
Sd3	553	431	221	97	4,481	609	162	9.6	36.3	59.8	5.66	4.23	3.30
Sd4	553	476	227	105	5,159	586	190	9.2	38.8	55.3	6.03	4.74	3.74

[a] Wb = desert soil, Sb = agricultural soil, Mc = *M. chamomilla*, Co = *C. officinalis*, Sd = *S. distichum*. Numbers indicate the independent replicate sample.

[b] quality reads were normalized to the same number of sequences.

[c] rarefaction curves are depicted in the Supplementary Figure S3.

Table S3. Relative composition of bacterial classes identified in the NifH sequence libraries of soils and medicinal plant rhizospheres at genus level.

	Desert Soil[a]		Agricultural Soil[a]	Closest database matches (GenBank accession number)	Similarity (%)
Alphaproteobacteria					
Rhizobium	93.7%		40.9%	*Rhizobium* sp. (GQ241353)	99-100%
Agrobacterium			12.9%	*A. tumefaciens* (FJ822995)	100%
Methylocystis			38.2%	*Methylocystis* sp. (EU934433)	100%
Bradyrhizobium	3.4%		5.1%	*B. elkanii* (AB501133)	95%
				B. japonicum (GQ289576)	99%
Mesorhizobium	1.6%			*M. loti* (AB367742)	98%
Methylocella			2.9%	*M. palustris* (AJ563948)	98%
Bacilli					
Paenibacillus			41.5%	*P. brasilensis* (EU294253)	96%
				Paenibacillus sp. (AJ223992)	95%
Cyanobacteria					
Anabaena			100%	*A. azotica* (DQ294218)	100%
	Matricaria chamomilla[a]	*Calendula officinalis*[a]	*Solanum distichum*[a]	Closest database matches (GenBank accession number)	Similarity (%)
Alphaproteobacteria					
Rhizobium	8.2-9.9%	8.2-28.5%	≤21.2%	*Rhizobium* sp. (GQ241353)	99-100%
Agrobacterium	≤17.1%	≤5.6%		*A. tumefaciens* (FJ822995)	96%
Ensifer	23.9-36.8%	18.1-57.1%	3.2-51.2%	*E. adhaerens* (JX081993)	97-100%
Methylocystis		≤3.0%	≤5.3%	*Methylocystis* sp. (AF378718)	99%
Bradyrhizobium	19.4-56.1%	18.0-39.0%	25.6-69.6%	*B. japonicum* (GQ289574)	95-97%
				Bradyrhizobium sp. (JX079649)	95-98%
				B. japonicum (GQ289566)	95%
Azospirillum	≤3.9%		≤11.5%	*A. amazonense* (GU256445)	100%
Betaproteobacteria					
Ideonella	≤58.6%	36.0-77.0%	22.2-93.5%	*Ideonella* sp. (AY231580)	97-99%
Burkholderia		11.0-34.7%	≤2.9%	*Burkholderia* sp. (JN247661)	98%
Derxia	≤47.8%	5.3-15.7%	≤3.6%	*D. gummosa* (AB089484)	100%
Dechloromonas	≤19.0%		1.9-65.3%	*Dechloromonas* sp. (AJ563286)	100%
				Dechloromonas sp. (JX154811)	100%
				Dechloromonas sp. (JX154844)	98%
Azoarcus		≤22.7%	2.6-9.2%	*A. communis* (U97116)	99%
				Azoarcus sp. (EF158389)	100%
Azospira			≤6.7%	*A. oryzae* (U97115)	98%
Zoogloea	≤100%	≤5.5%		*Z. oryzae* (AB201045)	98%
Azonexus			≤3.3%	*A. hydrophilus* (EF626686)	99%
Gammaproteobacteria					
Azomonas	100%		64.3-100%	*A. macrocytogenes* (AY644349)	99%
Deltaproteobacteria					
Geoalkalibacter			100%	*G. ferrihydriticus* (DQ660332)	98%
Bacilli					
Paenibacillus	≤100%			*P. brasilensis* (EU294253)	98%
				Paenibacillus sp. (AJ223992)	95%

[a] clusters with less than 1% of quality sequences were not designated.
